# Dysregulation of Nrf2 in Hepatocellular Carcinoma: Role in Cancer Progression and Chemoresistance

**DOI:** 10.3390/cancers10120481

**Published:** 2018-12-03

**Authors:** Azhwar Raghunath, Kiruthika Sundarraj, Frank Arfuso, Gautam Sethi, Ekambaram Perumal

**Affiliations:** 1 Molecular Toxicology Laboratory, Department of Biotechnology, Bharathiar University, Coimbatore 641 046, Tamilnadu, India; raghu_lifescience@hotmail.com (A.R.); krithisundarrajan@gmail.com (K.S.); 2 Stem Cell and Cancer Biology Laboratory, School of Pharmacy and Biomedical Sciences, Curtin Health Innovation Research Institute, Curtin University, Perth, WA 6009, Australia; frank.arfuso@curtin.edu.au; 3 Department of Pharmacology, Yong Loo Lin School of Medicine, National University of Singapore, Singapore 117600, Singapore

**Keywords:** hepatocellular carcinoma, Keap1-Nrf2-ARE pathway, microRNAs, Nrf2 dysregulation, phytochemicals

## Abstract

The liver executes versatile functions and is the chief organ for metabolism of toxicants/xenobiotics. Hepatocellular carcinoma (HCC) is the most common primary liver malignancy and the third foremost cause of cancer death worldwide. Oxidative stress is a key factor related with the development and progression of HCC. Nuclear factor erythroid 2 [NF-E2]-related factor 2 (Nrf2) is a cytosolic transcription factor, which regulates redox homeostasis by activating the expression of an array of antioxidant response element-dependent genes. Nrf2 displays conflicting roles in normal, healthy liver and HCC; in the former, Nrf2 offers beneficial effects, whereas in the latter it causes detrimental effects favouring the proliferation and survival of HCC. Sustained Nrf2 activation has been observed in HCC and facilitates its progression and aggressiveness. This review summarizes the role and mechanism(s) of action of Nrf2 dysregulation in HCC and therapeutic options that can be employed to modulate this transcription factor.

## 1. Introduction

Hepatocellular carcinoma (HCC) is the one of the leading liver cancers in many countries and is the fifth most common cancer worldwide [1]. Though liver cirrhosis contributes to 80–90% of HCC development, other factors, viz aflatoxin B1, diabetes, dietary habits, excessive alcohol intake, infection with Hepatitis-B-virus (HBV) and Hepatitis-C-virus (HCV), iron accumulation, non-alcoholic fatty liver disease and tobacco use also result in HCC development [2]. Many of these factors modulate the redox homeostasis in the liver, eventually causing hepatocarcinogenesis [3]. The disparity in the production of undue reactive oxygen species (ROS) and their disposal ends in oxidative stress. The Kelch-like ECH-associated protein 1 (Keap1)-nuclear factor E2-related factor 2 (Nrf2)-antioxidant response elements (ARE) pathway protects the cell against oxidative stress [4]. Nrf2, a CNC-bZIP transcription factor, regulates a prodigious number of genes, that can regulate redox status, detoxification of toxicants and transport of drugs. Thus targeted modulation of Nrf2 can be employed to treat various chronic diseases including cancer. On the contrary, the sustained enhancement of Nrf2 may also promote the survival and progression of cancer [5]. This review briefly summarizes the molecular structure, role and potential regulation of Nrf2 and also elaborates upon potential pharmacological strategies that can be used to target this transcription factor in cancer.

## 2. Epidemiology of HCC

**Figure 1:**
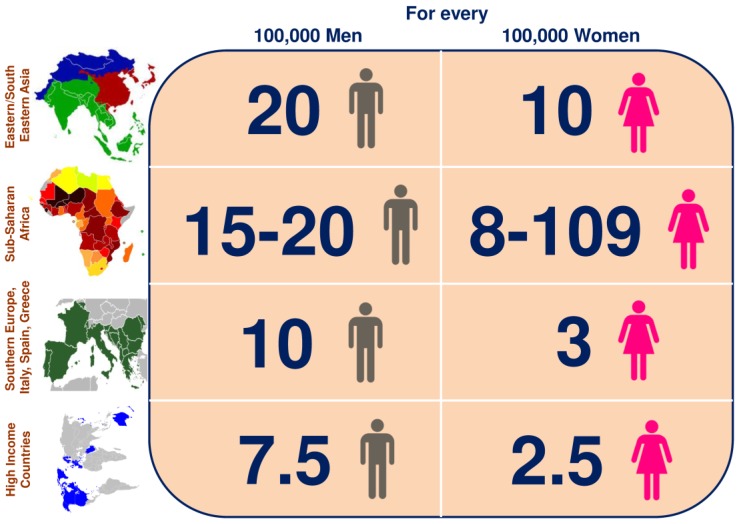
HCC around the world. Incidence rates of HCC in Eastern/South Eastern Asia, Sub-Saharan Africa, Southern Europe, Italy, Spain, Greece and high-income countries per 100,000 men and women.

The occurrence of HCC differs based on geographical regions [6,7] (Figure 1). HCC causes high impermanence and poor long term survival leading to a consequential worldwide health implications. According to the report in cancer facts and figures, the 5-year relative survival rate is 18% for liver cancer patients diagnosed with all stages. The 5-year survival rate increases to 31% when 43% of patients are diagnosed with a localized stage of disease [8]. Hepatitis-B-virus (HBV) and Hepatitis-C-virus (HCV) infections are the most primary risk factors for HCC development. HBV and HCV infections lead to HCC development, with an incidence of around 80% in Africa/East Asia and 20% in Western Europe and North America. Globally more than 400 million people are persistently infected with HBV and around 804/100,000 men and 178/100,000 women are HBV positive [9]. 170 million people worldwide are chronically infected with HCV and around 80% of HCC patients in low and intermediate income countries are HCV positive.

The various causative factors underlying HCC development include alcohol drinking, aflatoxin exposure, tobacco smoking, obesity, diabetes and genetic factors [10]. Fungal species produce carcinogenic aflatoxins that contaminate food, which then induces liver damage [11]. Sub-Saharan Africa, Southeast Asia and China are the most affected regions of aflatoxin contamination [12]. Similarly, chronic alcohol intake is a main risk factor for HCC development, with an incidence rate of 0.01/100 people with cirrhosis. Less than 40 g/day of alcohol is considered as a moderate alcohol intake and 40–60 g/day is considered as heavy alcohol drinking. Alcohol drinkers who are HBV and HCV positive have a higher risk of developing HCC compared with non-drinkers [13].

In Western societies, inflammation with non-alcoholic fatty liver disease leads to the development of HCC, along with obesity and type 2 diabetes mellitus [14,15]. Among 30–40% of patients with obesity-related metabolic changes are at risk of developing HCC worldwide [13]. Type 2 diabetes mellitus patients with cirrhosis, HCV infection, chronic liver disease and HBV infection generally exhibit a relatively higher risk of HCC [16]. Tobacco smokers may also have a 1.5 time’s higher risk of developing HCC than non-smokers [17].

## 3. Molecular Pathogenesis of HCC

Hepatocarcinogenesis is a multistep process, which involves alterations in gene expression pattern associated with mutations in liver-specific genes. These genes are associated with cell proliferation, cell cycle progression, apoptosis and metastasis, which include *p53*, adenomatous polyposis coli (*APC*), breast cancer 1 (*BRCA1*), breast cancer 2 (*BRCA2*), *Rb*, *Ras* and *β-catenin* [18]. Activation of growth factor regulating signalling pathways (insulin- like growth factor (IGF), epidermal growth factor (EGF), hepatocyte growth factor (HGF)), cell differentiation related pathways (Wnt, Hedgehog, Notch) and angiogenesis-induced signalling pathways (vascular endothelial growth factor) also promotes HCC pathogenesis [19].

Additionally, mutations and epigenetic alterations can also contribute to carcinogenesis. Specifically, 30–60% of HCCs are reported to exhibit mutations in the telomerase RNA component (*TERC*) and the telomerase reverse transcriptase (*TERT*) [20]. Consequently, down regulation of *p53* or *p53* mutation activates aggressive oncogenic pathways as well as development of HCC [21]. Accumulation of specific mutations in codon 249 of the seventh exon in *p53* inhibits cellular apoptosis and induces the proliferation of tumour cells. Deregulation of p14/ARF, growth arrest and DNA damage inducible protein (GADD45) are related with a high level of chromosomal instability with epigenetic alterations [22]. Special cases of HCC are mainly caused by site-specific hypomethylation and site-specific hypermethylation. In HCC, DNA methylation in specific gene regions results in genetic instability and silencing of tumour suppressor genes. HBV and HCV primarily target the DNA methyltransferases (*DNMTs*), *GSTP1*, *E-Cadherin* promoters and Ras/Raf/ERK signalling pathway genes [23,24].

Similarly, signalling pathway deregulation has also been attributed to HCC development. Mainly, Wnt/β-catenin, Ras, p14ARF/P53, p16INK4A/Rb, transforming growth factor-β (TGF-β) and PTEN/Akt signalling pathway deregulation is commonly associated with the development of HCC [25]. The Wnt/β-catenin signalling pathway is involved with cellular proliferation, development and motility and an overactivated Wnt/β-catenin pathway is involved in the development of human cancer [26]. Hyperactivation of the Ras/mitogen-activated protein kinase (MAPK) and phosphatidylinositol 3-kinae (PI3K)-Akt kinase signalling pathways has been observed in 50% of HCC [27]. Currently, TGFβ appears to play a significant role in cellular growth suppression in the initial stages as well as invasiveness in the final stages of HCC development [28]. However, HCC arises with the JAK/STAT pathway activation through inactivation of suppressors of cytokine signalling (SOCS) and signal transducers and activators of transcription (STAT)-induced STAT inhibitor 1(SSI-1) [29].

Recent research revealed that Nrf2 target genes can also play a potential role in hepatic injury, inflammation and hepatocarcinogenesis. HCV-induced hepatocarcinogenesis can involve the phosphorylation and nuclear translocation of Nrf2 by MAPK, casein kinase 2 and PI3K, which regulate the *Nrf2-ARE* genes by causing up regulation of Maf proteins [30]. However, HBV infected cells can also elicit up regulation of the Nrf2-regulated proteasomal subunit PSMB5 and cause a down regulation of immunoproteasome subunit PSMB5i [31]. Consistent with this, HepG2 cells expressing CYP2E1 showed an up-regulation of Nrf2 mRNA as well as protein expression and modulates the expression of various downstream genes (*GCLC* and *HO-1*) [32].

## 4. Oxidative Stress and Inflammation in HCC

Oxidative stress can be one of the major causes underlying HCC development, by driving DNA damage, reactive oxygen species (ROS), reactive nitrogen species (RNS) production and altered protein expression. Oxidative stress can cause mitochondrial damage to reduce the electron transport chain and is associated with an elevated level of ROS, tumour necrosis factor alpha (TNF-α) production, mitochondrial respiratory chain damage and cytochrome *c* oxidase impairment [33]. Specific causes of oxidative stress in the liver trigger chronic inflammation in hepatic stellate cells (HSCs), dendritic cells (DCs), Kupffer cells (KCs) and liver sinusoidal endothelial cells, with the suppression of cytokine production (Interleukin-(IL)-6) and cellular apoptosis [34]. These hepatic inflammation-induced superoxide anions are transformed by superoxide dismutase to produce high levels of H_2_O_2_ in hepatocytes.

**Figure 2:**
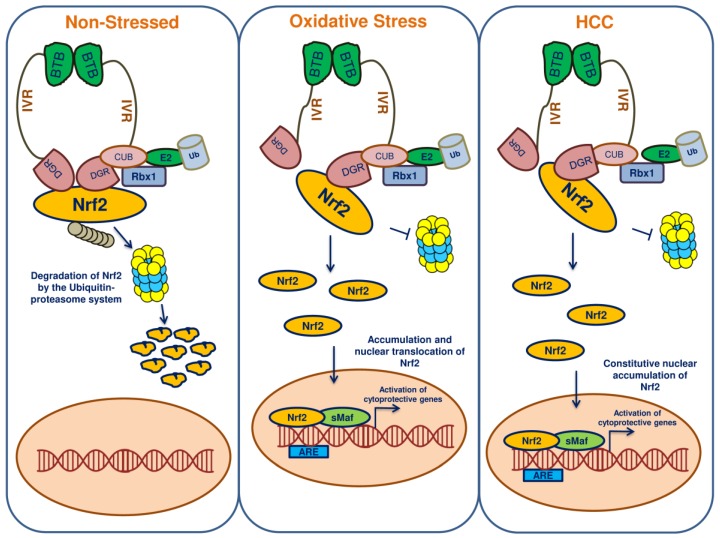
Keap1-Nrf2-ARE pathway in non-stressed, oxidative stress state and HCC cells. Keap1 has three characteristic domains: BTB, IVR and DGR domains. In the unstressed condition, Keap1 promotes Nrf2 degradation so that the level of Nrf2 remains very low. During oxidative stress, Nrf2 gets activated to maintain redox homeostasis. In HCC, sustained Nrf2 activation leads to cellular proliferation and resistance against drugs.

Toxic compound/carcinogenic agent exposure causes oxidative stress in hepatocytes and augments the translocation of Nrf2 into the nucleus. The Nrf2 expression is up regulated under the oxidative stress condition and thus regulates proliferation, survival and invasion processes in the tumour cells [35]. Zhang et al. [36] reported that Nrf2 promoted the proliferation and metastasis of HCC through the diminution of the apoptotic signalling pathway (Figure 2). HCV infections can negatively regulate the expression of Nrf2-ARE regulated genes to induce oxidative stress markers, leading to the viral hepatitis. Moreover, HCV-induced hepatotoxicity increased the accumulation of Nrf2 in a time-dependent manner, resulting in the transcriptional activation of Nrf2-ARE related genes associated with PI3K, casein kinase 2 and MAPKs expression [37].

Specific ROS may be generated by the direct effect of the β-oxidation pathway, leading to the leakage of electrons in the mitochondrial electron transport chain [38]. Recently, HSCs and KCs had a higher response to oxidative stress, which can directly lead to nuclear factor kappa B (NF-κB) activation that resulted in NO, ROS, cytokine synthesis and necrosis [39]. Increased ROS can induce more mitochondrial membrane permeability and DNA damage that has been found to be associated with deletion and mutation of apoptosis-specific genes in HCC [40]. HBV/HCV infection-induced oxidative stress causes specific gene mutations in the diverse processes of cell cycle, DNA repair and apoptosis [41]. Chronic integration of HBV into the genetic material may also result in accumulation of cancer-specific gene mutations. HBV infection can cause an increased release of IL-1β, IL-6, CXCL8 and TNF-α via NF-κB involvement [42]. The HBV multifunctional protein (called HBx) accumulates into the hepatocyte mitochondria to generate ROS, which results in the activation of the MLK3/MKK7/JNKs signalling pathways [43,44]. Similarly, HCV antigen deposition is associated with disruption of the immune system, with the resultant production of hydroxyl radicals, toxic ROS, perforin, granzyme B, inflammation and fibrosis [45]. Oxidative stress through HCV infection results in hepatocyte DNA mutation, infinite regeneration cycles and hepatocyte apoptosis [46]. Sequence variations in patatin like phospholipase domain containing 3 (PNPLA3) might lead to hepatic steatosis and liver injury. The polymorphism in PNPLA3 has been also reported to be associated with HCC [47].

The circulating cell-free DNA (cfDNA) are 160–180 base pair fragments, with the major contributor of this cfDNA being white blood cells. Circulating cell-free tumour DNA is released into the blood stream from the tumour cells undergoing apoptosis and necrosis. This circulating DNA could serve as a biomarker for the detection of metastasis [48]. Better clinical management of HCC can be made possible through determining the quantity and methylation status of cfDNA. The role and potential use of cfDNA in the diagnosis, management and screening of HCC has been reviewed in detail [49]. Similarly, microRNA (miR) can cause an inflammatory process and oncogenesis [50]. Oxidative stress is one of the important events that effectively down regulates the miRs (miR-92, miR-199a, miR-199b and miR-122a) in the immune system and directly causes HCC [51].

## 5. Structure of Nrf2

**Figure 3:**
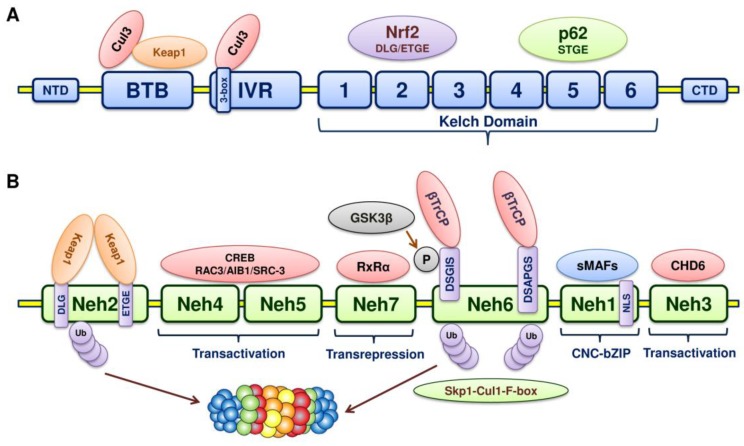
(**A**) Domain structure of Keap1. The BTB domain mediates homodimerization with another Keap1 and associates with Cul3. The IVR domain also mediates interaction with Cul3 and is a redox sensor due to the presence of cysteine residues. The Kelch domain is essential for Nrf2 and p62 (Sqstm1) binding. (**B**) Domain structure of Nrf2. The N-terminal Neh2 domain has DLG and ETGE motifs, which are essential for Keap1 binding. The transactivation activity of Nrf2 is mediated through Neh4, Neh5 and Neh3. Neh7 interacts with RxRα and is important for transrepression. Neh6-a redox insensitive degron domain-has DSGIS and DSAPGS motifs, which mediate Keap1-independent proteasomal degradation of Nrf2. GSK3β phosphorylates the DSGIS motif, which brings β-TrCP into action for subsequent ubiquitination and proteasomal degradation. Neh1 has a bZIP motif that binds AREs upon dimerization with sMafs.

The *NFE2L2* gene encodes a cap “n” collar (CNC) basic-region leucine zipper (bZIP) transcription factor, Nrf2 [52,53]. Nrf2 (66-kDa) consists of 7 Nrf2-erythroid cell-derived proteins with CNC homology (ECH) domains with 605 amino acids (Neh 1–7); each of the Neh domains executes distinct functions. The Neh1 domain is essential for two functions: One being DNA binding by the CNC-bZIP region and the other is dimerization with other transcription factors [54] (Figure 3). The amino acid residues of the Neh1 domain are basic and hence facilitate the binding with acidic DNA. This Neh1 domain is conserved across different species and thus distinguishes itself as an important functional domain within Nrf2. In addition, Nrf2 stability is mediated by the Neh1 domain on interaction with a conserved metazoan E2 ubiquitin conjugating-enzyme (UbcM2) [55]. When disengaged from the Keap1 protein complex, the bipartite nuclear localization signal in the Neh1 domain gets uncovered so that the translocation of Nrf2 happens.

The major regulatory N-terminal Neh2 domain interacts with Keap1 through two highly conserved motifs: ETGE and DLG [56,57]. Both these motifs bind the Kelch domain of Keap1. The ubiquitination of 7 lysine residues in this Neh2 domain directs Nrf2 for proteasomal degradation, thus Neh2 is mandatory for negative regulation.

The transcriptional activation of Nrf2 regulated genes is executed by Neh3, Neh4 and Neh5 domains along with their coactivators [58]. The Chromo-ATPase/helicase DNA-binding protein family member, CHD6, facilitates the activation of transcription by binding with the Neh3 domain [59].

Similarly, a CH3 domain of coactivator cyclic adenosine monophosphate (cAMP)-responsive element-binding (CREB) protein triggers Nrf2 transactivation through interaction with both the Neh4 and Neh5 domains [58]. Both these Neh4 and Neh5 transactivation domains elevate the Nrf2-targeted ARE genes’ expression in association with nuclear cofactor receptor-associated coactivator 3 (RAC3)/AIB-1/steroid receptor coactivator-3 (RAC3/AIB1/SRC-3) [60]. The cellular localization of Nrf2 is regulated by the Neh5 domain, which possesses a redox-sensitive nuclear export signal.

The Neh6 domain carries two important motifs: DSGIS and DSAPGS, which are highly conserved and the β-transducing repeat-containing protein (β-TrCP) recognizes these two motifs [61]. Glycogen synthase kinase-3β (GSK3β) phosphorylates the DSGIS motif, which makes binding of β-TrCP to Neh6 more efficient. GSK3β directs the proteasomal degradation of Nrf2 independent of Keap1, with the recruitment of the Skp1-Cul1-F-box (SCF) protein ubiquitin ligase complex [62,63,64]. As the degradation is independent of Keap1, the Neh6 domain has been designated as the redox-insensitive region of Nrf2 [65].

The retinoid X receptor alpha (RxRα)-an Nrf2 repressor-associates with the Neh7 domain to inhibit the transcriptional activity of Nrf2 [66].

## 6. Structure of Keap1

Once scientists had strong evidence that a cellular factor can negatively regulates Nrf2, the quest for identification of the negative regulator began. Nrf2 repression was controlled by a single conserved domain-Neh2-in Nrf2. The location of this specific Neh2 domain led to the discovery of Keap1-a new negative regulator [56]. Keap1 binds this N-terminal Neh2 domain and represses Nrf2. It was named Keap1 due to its similarity with Drosophila Kelch-a cytoskeleton binding protein.

Keap1 is a Kelch-like (KLHL) protein, which belongs to the family of BTB-Kelch proteins. Keap1 complexes with Rbx1 and Cullin 3 (Cul3) and forms multicomplex Cullin-really interesting new gene (RING) ligases (CRLs) for polyubiquitination and subsequent degradation of Nrf2; thereby preventing Nrf2 translocation into the nucleus and binding of Nrf2 with antioxidant response elements (AREs) in the DNA. Similar to KLHL protein, Keap1 has three main domains with an N-terminal region (residues 1–49), a Broad complex, Tram-track, a Bric a brac (BTB) domain (residues 50–179), an intervening region (IVR) with residues 180–314, the double-glycine repeat or Kelch domains and carboxy terminal region (CTR) (residues 327–611) [67,68]. Being a substrate adaptor, with its BTB domain, Cul3 and Nrf2 are linked, the IVR domain binds Cul3 and the Kelch domain binds motifs in the Neh2 domain of Nrf2 (Figure 3).

The BTB domain (aka POZ domain) is responsible for the both homo- and heterodimerization [69]. The N-terminal BTB domain is essential for dimerization. It docks the Cul3-dependent E3 ubiquitin ligase complex [70,71]. The IVR domain (aka BACK domain) mediates the interaction of Cul3 with Keap1 through the 3-box bihelical motif in the proximal part in the N-terminal end called the ‘3-box’ [72]. The IVR cysteine residues act as sensors of electrophiles and ROS and thus play an essential role in redox homeostasis. The IVR domain connects the BTB and the Kelch domain.

The Kelch domain (aka DGR or DC domain) consists of 6 Kelch repeats forming 6 β-propeller blades (I-VI). Each blade possesses 4-antiparallel β-sheets (beta strands A–D) [73]. Loops of different lengths link these 4 β-strands as the loops arise from the β-propeller central core. The bottom of the β-propeller has long loops linking β-strands B to C and D to A, while the top of the β-propeller has short loops joining β-strand A to B and C to D. The central portion of the propeller forms a channel that runs through the entire domain. The conserved amino acid residues in the Kelch domain include a tyrosine (βC), a tryptophan (βD) and a double-glycine repeat (DGR) and hence are named the DC and DGR domain [74]. The Keap1 DGR domain is vital for the interaction with the Neh2 domain of Nrf2, whereas the tyrosine and tryptophan are needed for the hydrophobic packing between blades [73]. The Kelch domain holds Keap1 in the cytoplasm via interaction with the actin cytoskeleton [75]. The Keap1 binds the N-terminal Neh2 domain of Nrf2 with the Kelch domain [76]. The Kelch domain of two Keap1 units binds to the Neh2 ETGE motif with high affinity and DLG motifs with low affinity separately [77]. The Kelch domain of one Keap1 sequesters the Nrf2 in the cytoplasm with the ETGE motif, whereas the DLG motif of the other Keap1 locks Nrf2. In an unstressed state, two Kelch domains sequester Nrf2 on binding with the Glu-Thr-Gly-Glu (ETGE) and Asp-Leu-Gly (DLG) motifs on the Neh2 domain [78].

The disruption of Keap1 can also lead to elevated cellular Nrf2 activity. This disruption happens when somatic mutations occur in Keap1 [79,80], Keap1 translational repression or mRNA degradation by miRs [81,82] and hypermethylation of cytosine in the Keap1 promoter [83]. All these occur in human cancer and hence Keap1 may act as tumour suppressor. Moreover, as a result of Keap1 disruption, constitutive Nrf2 activation can lead to drug and radiation resistance in tumours [84].

## 7. Keap1-Nrf2-ARE Pathway

Nrf2 is the primary governor of the antioxidant response pathway [85]. During normal physiological conditions, Keap1 acts as a negative regulator of Nrf2 and maintains a basal level of Nrf2 in the cytoplasm by degradation through the ubiquitin-proteasomal system. In contrast, the level of Nrf2 has been reported to be elevated during the stress conditions, primarily during oxidative stress. The Keap1 IVR domain cysteine residues act as redox sensor and when they get oxidized, the Nrf2 is disengaged from the regulatory complex [86]. Then, Nrf2 becomes phosphorylated and translocates into the nucleus as the nuclear localization signal sequences are exposed. Within the nucleus, the prime dimerizing partners-small v-maf musculoaponeurotic fibrosarcoma oncogene homolog (sMaf) proteins-accompany Nrf2. The Nrf2-sMaf heterodimers bind specific AREs within the DNA, which results in the recruitment of other transcriptional activators and triggers the transcription of Nrf2 target genes [87,88]. In HCC, this pathway may be enhanced and promotes cellular proliferation and progression. Thus, Nrf2 has been considered both as a tumour suppressor and an oncogene [89]. As it protects the cell from stress insults, Nrf2 is considered to be a tumour suppressor [90]. But during the initiation of cancer, oxidative stress persists, leading to the hyperactivation of Nrf2, which can then promote the survival of cancer cells [91]. During the course of its action in cancer cells, Nrf2 may also confer cellular resistance against chemotherapeutics and radiation therapy.

## 8. Nrf2 and Keap1 Mutations Lead to HCC

ROS generated by either endogenous metabolites or exogenous toxicants cause mutations in key genes to initiate cancer. Aberrant Nrf2 activation occurs due to somatic mutations in Keap1 and Nrf2, which can lead to the disrupted binding between Keap1 and Nrf2. Driver mutations offer a selective growth advantage and can promote cancer. Recently a study identified 30 candidate driver genes from 503 liver cancer genome data [92]. Most of these genes encode activators of the mammalian target of rapamycin (mTOR) pathway, chromatin remodellers and metabolic enzymes. Yet another study identified point mutations, structural variations and integrations of viral DNA in coding and non-coding regions in 300 liver cancers from Japanese individuals [93]. The frequency of mutations in Nrf2 (5.1%) is greater than Keap1 (3.2%) in HCC [94]. The mutations in Keap1 and Nrf2 occur as late events in HCC, as observed in the advanced stage of human liver carcinogenesis [95]. In contrast, the mutations in Nrf2 occur as an early event in the resistant hepatocyte model, leading to carcinogenesis [96]. In the case of Nrf2, most of the mutations fall in the low affinity DLG and high affinity ETGE motifs of the Neh2 domain in HCC [96,97]. Yet, mutations in the DLG motif can induce the activation of Nrf2. The loss-of-function Keap1 mutations induce persistent activation of Nrf2. Next generation sequencing data indicated that 6% of HCC are caused by mutations/ROS induced oxidative stress conditions. Dissociation of Keap1 and Nrf2 by oxidative stress increases the nuclear translocation of Nrf2, which thereby regulates the expression of antioxidant genes and [98].

It has been reported that 78.6% of Nrf2/Keap1 mutations occur in early HCC, 59.3% mutations in advanced HCC and 71% in early pre-neoplastic lesions. The mutation of Keap1 has a lower frequency compared to Nrf2 in HCC and preneoplastic lesions [93]. Nault and Zucman-Rossi [99] reported that 8% of mutations were identified in *Keap1* and the NFE2L2-Keap1 pathway and were associated with high transcript levels. Specifically, the Nrf2 DLG motif contains 80% of its mutations in codons encoding amino acid residue 29 and 100% of its mutations in amino acid residue 32 [100]. A recent study reported that Nrf2 mutations were increased in the specific motif LxxQDxDLG (spanning amino acid 17–32) or DxETGE (amino acid 77–82) that binds the Kelch domain in Keap1. These mutations easily up regulate the Nrf2 target genes in proneoplastic lesions [96].

## 9. Nrf2 in HCC

Hepatocytes that are exposed to chemical carcinogens can accumulate ROS, leading to cellular oxidative stress and resulting in hepatocyte transformation of HCC progenitor cells [101]. The transient activation of Nrf2 offers protection during this process but in contrast, persistent activation of Nrf2 can drive the oncogenic process. The use of antioxidants as Nrf2 activators will lead to the sustainable activation of Nrf2, thereby contributing to carcinogenesis. Hence, a promising therapeutic strategy is the use of Nrf2 inhibitors, which might deter the progression of chronic liver damage to HCC.

In HCC, Nrf2 expression has been found to be related with survival of the cells and clinicopathological factors [36]. The increased expression of Nrf2 was corroborated with differentiation, metastasis and size of the tumour in HCC. In addition to Nrf2 expression, the proliferation and invasion of HCC cells were evidenced by the expression of Bcl-xL and matrixmetallaproteinase-9. Hence, Nrf2 expression could be utilized as an independent prognostic factor in HCC. An interesting observation was made in Hepa-1 and HepG2 cells where Nrf2 mediated the expression of Bcl-xL with AREs in the promoter region of Bcl-xL [102]. This Bcl-xL expression induced by Nrf2 prevented apoptosis and promoted drug resistance as well as survival in both the cell lines. In HCC patients, increased Nrf2 expression and 8-hydroxyguanosine (8-OHdG) lesions were observed [103]. This elevated 8-OHdG was found to be associated with poor survival. Similar observations were also made in HepG2 cells on exposure to H_2_O_2_.

In Nrf2-deficient mice, chronic exposure to non-genotoxic hepatocarcinogens-pentachlorophenol (PCP) and piperonyl butoxide (PBO)-triggered oxidative stress [104]. This oxidative stress can stimulate the proliferation and progression of preneoplastic lesions to neoplasms. This is indicative of Nrf2 dysregulation increasing the risk of carcinogenesis elicited by non-genotoxic environmental carcinogens.

The presence of high affinity enhancer element (GPE1) in the placental glutathione S-transferase (GST-P) regulates the expression of GST-P through Nrf2-MafK [105]. The Nrf2-MafK heterodimer can also drive the expression of GST-P in early hepatocarcinogenesis and in HCC but not in normal liver cells.

## 10. Autophagy, p62 and Nrf2 in HCC

An interesting observation was made in Japanese HCC, where phosphorylation of p62 (aka sequestome-1) conferred Nrf2 activation in contrast to non-tumour regions [106]. The phosphorylation of p62 and its contribution to tumorigenesis has to be further investigated. Yet, another study has reported that p62 expression contributed to the resistance of HCC against ferroptosis [107], which is a form of regulated cell death induced by lipid ROS generated by iron-species [108]. Erastin, sorafenib and buthionine sulfoximine can induce ferroptosis in HCC. p62 activation brings in resistance against these compounds in HCC. p62 activated Nrf2 by degrading Keap1, which eventually triggered the transcription of Nrf2-target genes-*Nqo1*, *HO-1* and *FTH1*. These Nrf2-target genes reduce the lipid peroxidation and regulate iron homeostasis in HCC, thereby conferring resistance against ferroptosis. This p62-Keap1-Nrf2 pathway activation can mediate resistance of HCC against ferroptosis inducing compounds. Hence, Nrf2 inhibitors in combination with ferroptosis inducers may serve as a promising therapeutic strategy for liver cancer.

**Figure 4:**
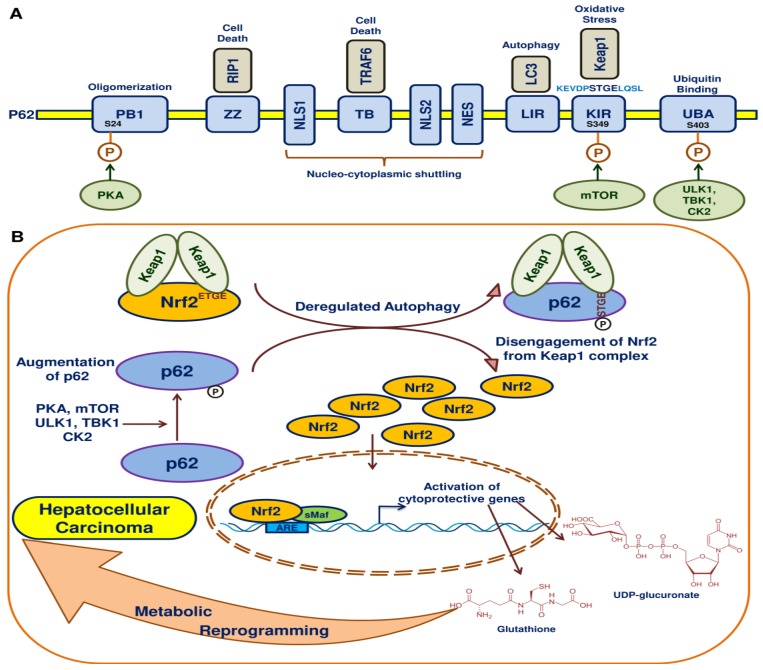
Autophagy, Keap1 and p62 regulate Nrf2 in HCC. (**A**) Domain structure of p62/Sqstm1. The domains of p62 and their interacting partners. (**B**) Under normal physiology, Nrf2 is held by Keap1 and directs Nrf2 for proteasomal degradation. During deregulated autophagy, augmentation of p62 occurs through phosphorylation of p62. Upon phosphorylation, p62 sequesters Keap1 and disengages Nrf2 from Keap1, leading to the activation of the Keap1-Nrf2-ARE pathway, which results in antioxidant defence, survival and metabolic reprogramming in HCC.

Relentless Nrf2 activation with the accumulation of p62 was identified in autophagy-deficient mice and in human HCCs [109]. The homeostatic level of p62 is the key in the pathogenesis of liver injury, where autophagy regulates the p62 levels [110]. The cytoplasmic inclusion body formation is under the control of p62 levels. The deficient autophagy leads to liver injury through intracellular inclusion body formation. The selective autophagy substrate p62 can accumulate during the suppression of autophagy and activate Nrf2 [111]. This increased p62 leads to the competition of p62 with the binding of Nrf2 with Keap1. The p62-Keap1 complex aggregates in turn can stabilize Nrf2 and the activated Nrf2 binds AREs and triggers the transcription of Nrf2 target genes. During oxidative stress, p62’s affinity with Keap1 increases the phosphorylation of p62 and eventually competes with Nrf2’s binding with Keap1. This suppression of Keap1 augments the activation and translocation of Nrf2 into the nucleus, where Nrf2 activates genes to reprogram redox homeostasis and thus promotes the proliferation of HCC. This activation of p62 occurs when the autophagic process is deregulated in HCC cells (Figure 4). An interesting observation was made that p62 is essential for the induction of HCC in mice during preneoplasia [112]. This elevated expression of p62 enables the HCC-initiating cells to survive from stress and also protects them from oxidative damage induced cell death.

An impediment in autophagy induces post-translational modifications in p62 such as mTOR phosphorylation of S349 in the STGE motif (KIR domain) of p62, PKA phosphorylation of S24 of the PB1 domain of p62 and unc-51-like kinase 1 (ULK1), TANK-binding kinase 1 (TBK1) and casein kinase 2 (CK2) phosphorylation of S403 in the UBA domain of p62 [113]. All these phosphorylation events in p62 augment p62-Keap1 binding, ultimately resulting in the activation of Nrf2. Though these phosphorylation events are reported in different studies, the mode of activation of p62 in HCC was not studied in detail. Such future investigations will shed light on new therapeutic targets in HCC. The role of the Nrf2-p62 pathway in HCC has previously been reviewed in detail [114].

Similarly, increased phosphorylation of p62 advanced malignancy in HCV positive HCC patients [115]. Through Nrf2 activation, phosphorylation at S349 in p62 enhanced the synthesis of glutathione and UDP-glucuronate from glucose through the glucuronate pathway. HCV positive HCC patients provided evidence that p62 activation of Nrf2 regulates metabolic pathways, eventually promoting malignancy. Besides accelerating cellular proliferation, GSH brings in tolerance against chemotherapeutics by exporting them into the extracellular space through multidrug resistance (MDR) proteins. The development of MDR in HCC through the activation of Nrf2 has been reviewed elsewhere [116]. Metabolic reprogramming is the key event elicited by the p62-Keap1-Nrf2 pathway in HCC. Therefore, drugs that target phosphorylated p62 could be a therapeutic agent in HCC. K67 was identified in a chemical screen to disrupt the interaction between phosphorylated p62 and Keap1, which reinstated E3-ligase adaptors and eventually directing ubiquitination and degradation of Nrf2 by Keap1 in HCC cells [115]. Thus, K67 inhibited cellular proliferation and promoted susceptibility to anti-cancer drugs in HCC.

## 11. Phytochemicals/Molecules Can Elicit Activation of Nrf2 in HCC

**Figure 5:**
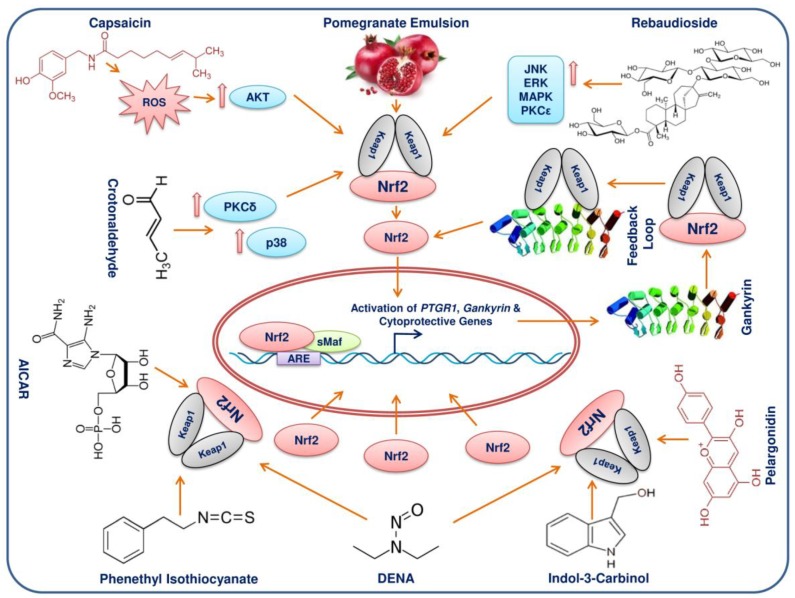
Phytochemicals and molecules that activate Nrf2 in HCC. Phytochemicals trigger the transcriptional activation of Nrf2 target genes by activating Nrf2 through different pathways.

Phytochemicals and other molecules display the potential to elicit Nrf2 activation in HCC directly or through other signalling mechanisms (Figure 5). Gankyrin (gann ankyrin-repeat protein) is the p28 component of the 26S proteasome and this seven ankyrin-repeat protein is evidenced as an oncoprotein [117]. The increased expression of gankyrin was shown to be higher in HCCs than in non-cancerous hepatic tissues [118,119]. Oxidative stress is the prime factor that leads to hepatocarcinogenesis [3]. Gankyrin has been observed to protect the HCC cell from oxidative stress [120]. The positive feedback loop between Nrf2 and gankyrin was established in human HCC to sustain redox homeostasis. Under oxidative stress, gankyrin expression abrogates ROS through the activation and stabilization of Nrf2 [120]. Gankyrin competes with Nrf2 and binds with the Kelch domain of Keap1, eventually preventing the ubiquitination and degradation of Nrf2. Interestingly, the promoter of gankyrin carried AREs and as a result, Nrf2 activation not only maintained homeostasis but also triggered the expression of gankyrin through a feedback loop [120]. Thus, gankyrin connects the link between oxidative stress and HCC development. The disruption of the interaction between gankyrin and Keap1 may serve as an effective therapeutic strategy against HCC.

An AMPK activator- 5-aminoimidazole-4-carboxamide riboside (AICAR)- is metabolized into 5-aminoimidazole-4-carboxamide ribotide (ZMP) [121], which elicits Nrf2 activation in HCC [122]. The increased ROS reinforce cellular adaptation through AMPK and regulate redox homeostasis. During the knock down of Nrf2, AICAR fails to regulate redox homeostasis in HCC. While the AMPK is silenced, the Nrf2 still gets activated on treatment with AICAR in HCC. This activation of the Nrf2 pathway irrespective of AMPK activity revealed that AICAR and its metabolite ZMP triggers Nrf2 activation via a non-canonical pathway. The mechanism of activation of Nrf2 by AICAR requires further investigation to identify new players.

Prostaglandin reductase 1 (PTGR1), aka NADP+ -dependent leukotriene B4 12-hydroxydehydrogenase, is essential for prostaglandin and eicosanoid catabolism and responds to inflammation and antioxidants [123]. In both a human clinical and a diethylnitrosamine (DENA) induced hepatocarcinogenesis rat model, the expression of PTGR1 was elevated [124]. The increased expression of *Ptgr1* in hepatocarcinogenesis increased cellular proliferation with reduced time and prevented the apoptosis elicited through oxidative stress [125]. An interesting observation is that Nrf2 regulated the expression of *Ptgr1*, which carried an ARE upstream (−653) of the transcription start site. In addition to *Ptgr1*, the expression of other Nrf2 target genes *Gclc*, *Nqo1* and *Gstp1* was also elevated to curb the oxidative stress. Thus, stable expression of PTGR1 may reduce the proliferation time, enable apoptosis evasion and offer metabolic adaptation.

Capsaicin (*trans*-8-methyl-*N*-vanillyl-6-nonenamide) is a homovanilic acid derivative found in red chilli pepper and has chemoprotective effects [126]. Capsaicin treated HepG2 cells generated ROS, which on interaction with Nqo1 inhibited its enzyme activity. The resulting increased ROS activated the PI3K-Akt signalling pathway through the phosphorylation of Akt, leading to the activation of Nrf2 [127]. Nrf2 then increased the expression of HO-1 while capsaicin inhibited Nqo1 activity, leading to the increased generation of ROS in HepG2 cells.

Crotonaldehyde is a highly reactive α, β-unsaturated aldehyde and a main constituent of cigarette smoke and is also present in bread, cheese, fruits, milk, meat and vegetables [128]. In HepG2 cells, crotonaldehyde induced HO-1 expression through the activation and translocation of Nrf2 into the nucleus [129]. Crotonaldehyde triggered activation of the protein kinase C-δ and p38 mitogen-activated protein kinase pathway, which ultimately activated the Nrf2 pathway. HO-1 is the primary player in the crotonaldehyde activated HepG2 cell survival pathway to counteract apoptosis and could serve as therapeutic target in crotonaldehyde promoted tumour resistance. Another plant product, tigloylgomisin H (TGH), is a lignan from the fruit of *Schisandra chinensis* and displays the potential to activate and translocate Nrf2 into the nucleus [130]. TGH induced NQO1-a phase II detoxification enzyme-in Hepa1c1c7 and BPrc1 cell lines. Hence TGH could serve as a potential HCC preventive agent.

Rebaudioside (13-[(2-*O*-β-d-glucopyranosyl-3-*O*-β-d-glucopyranosyl-β-d-gluco pyra nosyl) oxy]kaur-16-en-18-oic acid β-d-glucopyranosyl ester) is a steviol diterpene glycosidic constituent from the *Stevia rebaudiana* Bertoni leaves [131]. Rebaudioside (Reb A) is a natural sweetener with 250 to 450 times more sweetness than sucrose and is used as a non-caloric sweetener in many countries [132]. Several pharmacological and toxicological properties of Reb A were reviewed recently [133]. Reb A displayed antioxidant activity in HepG2 cells [134], where it alleviated the oxidative injury induced by carbon tetrachloride (CCl_4_). Nrf2 activation elicited by Reb A was evidenced in CCl_4_-treated HepG2 cells. Oxidative damage was recuperated by HO-1 and NQO1 expression, followed by the activation of Nrf2. This enhancement of Nrf2 activation occurred through the upregulation of JNK, ERK, MAPK and PKCε signalling.

Citrinin [(3*R*,4*S*)-8-hydroxy-3,4,5-trimethyl-6-oxo-4,6-dihydro-3*H*-isochromene-7-carboxylic acid] is a mycotoxin and a contaminant in human foods. Citrinin is produced by fungal strains from the genera *Aspergillus*, *Monascus* and *Penicillium* [135]. Citrinin inhibited the proliferation of HepG2 cells through the generation of ROS, leakage of lactic dehydrogenase and depolarization of mitochondrial membrane potential and elicited apoptotic cell death due to DNA damage [136]. Pelargonidin is a natural polyphenolic anthocyanidin pigment found abundantly in berries and radishes [137]. Pre-treatment with pelargonidin chloride (PC) reduced the cytotoxicity induced by citrinin in HepG2 cells. Mechanistic insights revealed that PC enhanced the level and activity of detoxification enzymes through activation of the Keap1-Nrf2 pathway.

The pomegranate fruit from *Punica granatum* is called superfruit for its enormous amount of polyphenolic constituents and virile antioxidant properties [138]. Pomegranate juice offered protection against systemic oxidative stress in mice [139]. The pomegranate emulsion (PE) prevents rat hepatocarcinogenesis induced by DENA, which emulates human HCC [140]. The active constituents in the PE induced the transcriptional activation of hepatic antioxidant and carcinogen detoxifying enzymes in DENA treated animals through the activation of Nrf2. Oxidation of proteins and lipids gets attenuated with PE. Thus, active constituents present in the PE could act as cancer preventive agents.

Phytochemicals such as phenethyl isothiocyanate, indole-3-carbinol, protocatechuic acid and tannic acid increased the expression of phase II enzymes (GSTA, GSTP, GSTM, GSTT and NQO1) and antioxidant enzymes (CAT, SOD, GR and GPx) in HepG2 cells [141]. This phase II and antioxidant gene expression was increased through activation of the Keap1-Nrf2-ARE signalling pathway. The induction was far more effective with phenethyl isothiocyanate and indole-3-carbinol than protocatechuic acid and tannic acid.

*Camptotheca acuminata* produces a planar pentacyclic quinolone alkaloid called camptothecin, which possesses anti-tumour activities [142]. Camptothecin is a DNA topoisomerase I poison that complexes with type I DNA topoisomerase and stabilizes the complex, preventing the cleavage and relegation reactions during DNA replication that ultimately halt cellular proliferation. In recent years, camptothecin was identified as a potent Nrf2 inhibitor. In HCC cells (HepG2 and SMMC-7221) and in xenograft models, camptothecin inhibited Nrf2 expression and sensitized the cells to various chemotherapeutic drugs [143]. Camptothecin may enhance the efficacy of combinatorial therapy in cancers with elevated expression of Nrf2. All these phytochemicals and other molecules may modulate Nrf2 activation by targeting different molecules in HCC (Table 1).

## 12. Phytochemicals/Molecules Sensitizing Resistant HCC through Nrf2 Signalling

**Figure 6:**
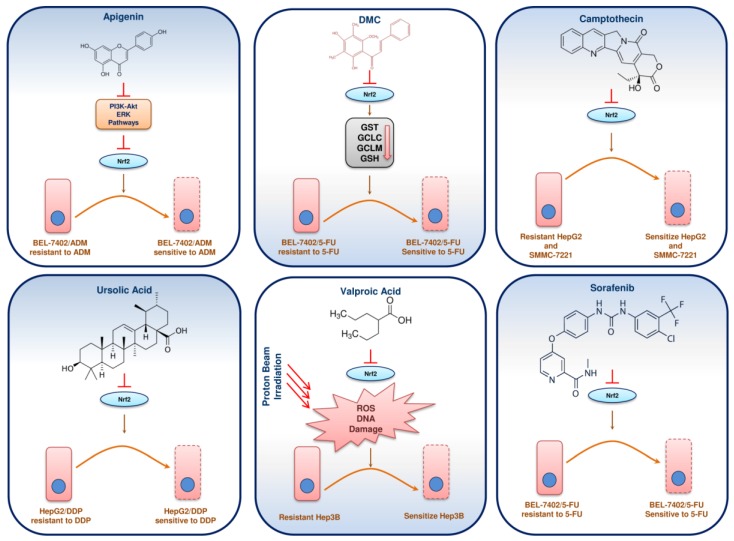
Phytochemicals/molecules that inhibit Nrf2, leading to the sensitization of drug resistant HCC.

Chemotherapy resistance is a common threat in the treatment of HCC. Phytochemicals and molecules have the potential to sensitize chemoresistant HCC cells (Figure 6). Apigenin (4′,5,7-trihydroxyflavone) is a natural bioflavonoid that has exhibited anti-cancer properties against various cancers. The sensitivity of doxorubicin resistant HCC (BEL-7402/ADM) was enhanced by apigenin at non-toxic concentrations [146]. The cytotoxicity of BEL-7402/ADM cells was increased when apigenin increased the intracellular concentration of adriamycin (ADM). The PI3K/Akt pathway offers chemoresistance to cancers through Nrf2 activation. Apigenin, as an effective adjuvant sensitizer with ADM, inhibits the PI3K/Akt pathway and its downstream Nrf2 pathway, thus sensitizing the BEL-7402/ADM cells to ADM. Yet another natural flavonoid, chrysin (5,7-dihydroxyflavone) sensitizes ADM resistant BEL-7402/ADM cells to doxorubicin through inhibition of the PI3K-Akt and ERK pathways [147]. The inhibition of these pathways ultimately suppresses Nrf2 expression and reverses the drug-resistant phenotype in BEL-7402/ADM cells.

2′,4′-Dihydroxy-6′-methoxy-3′,5′-dimethylchalcone (DMC) is a chalcone, which is an active ingredient found in the buds of *Cleistocalyx operculatus* and also an important ingredient of herbal tea [148]. The hepatoprotective effect and anti-tumour activity of DMC were reported in mice [149] and SMMC7721 HCC cells [150] respectively. The investigation on the anti-cancer mechanism of DMC in BEL-7402/5-FU cells revealed that DMC inhibited drug efflux by decreasing the intracellular GST activity and GSH content [151]. DMC, being an Nrf2 inhibitor, repressed the Nrf2 expression, leading to the down regulation of the downstream glutamate-cysteine ligase catalytic subunit (GCLC) and glutamate-cysteine ligase modifier subunit (GCLM). Hence, DMC could serve as an efficacious drug sensitizing agent against 5-FU resistant HCC.

Sorafenib (Nexavar) is a multi-kinase inhibitor, which is used as an anti-cancer agent, particularly against advanced HCC [152]. Sorafenib interferes with cell growth and invasion. The chemoresistance to 5-FU is a serious problem in HCC. Sorafenib reduces the 5-FU resistance in BEL-7402/5-FU cells, wherein the suppression of Nrf2 is the reverse mechanism [153]. Hence, sorafenib could be used as an Nrf2 inhibitor in HCC.

Ursolic acid (UA) is a lipophilic pentacyclic triterpenoid that imparts a smooth waxy appearance to fruits, particularly apples and is found in many fruits and herbs [154]. The reversal of resistance to cisplatin was brought about in cisplatin-resistant HepG2/DDP cells by UA [155]. The inhibition of Nrf2 signalling is the primary mechanism utilized by UA. Hence, UA can be utilized as a natural adjuvant sensitizer in cisplatin chemoresistant HCC.

Valproic acid (VPA)—a branched short-chain fatty acid, is the primary food and drug administration (FDA) approved drug for epilepsy and is a histone deacetylase inhibitor [156,157]. VPA sustained the proton beam generated ROS and DNA damage, eventually leading to apoptosis in Hep3B cells. VPA was shown to have a synergistic effect on proton beam irradiation [158]. The Nrf2 expression was suppressed not only in the Hep3B cells but also in Hep3B tumour xenograft models. Thus, VPA acts as a radiosensitizer in HCC targeting the Nrf2 signalling pathway. Investigations revealed that phytochemicals and other molecules have the potential to sensitize chemoresistant HCC through the suppression of Nrf2 (Table 2).

## 13. Endoplasmic Reticulum Stress and Nrf2 Signalling in HCC

The endoplasmic reticulum (ER) is the primary organelle that regulates multiple functions such as Ca^2+^ homeostasis, protein folding and trafficking, post-translational modification of proteins and lipid synthesis [159]. The microenvironment of normal tissues is different to that of the tumour microenvironment; the latter has hypoxia, low pH, lack of nutrients, physical pressure, immune surveillance and an imbalance between oxidants and antioxidants [160]. These factors perturb ER functions and can lead to ER stress in tumour cells [161]. In order to expand the tumours, ER stress can mediate to re-establish homeostasis and provides- ideal conditions for progression and survival [162]. When the ER stress response fails to restore homeostasis, apoptosis may be triggered. In HCC (HepG2, MHCC97L and xenograft), the elevated expression of fibroblast growth factor 19 (FGF19) and fibroblast growth factor receptor 4 (FGFR4) directs them to induce a cell survival response [163]. ATF4 -an ER stress inducible transcription factor- regulates the expression of FGF19 by binding to the promoter of FGF19. Further investigation from FGFR4 knockout studies revealed that FGFR4 is involved in the resistance to ER stress. The FGF19-FGFR4 axis ultimately induced Nrf2 accumulation through GSK3β. The phosphorylation at specific residues of GSK3β led to the inhibition of GSK3β and facilitated Nrf2 nuclear accumulation, which eventually offered cytoprotection. Thus, targeting the FGF19-FGFR4 axis might also aid in the treatment of HCC.

## 14. Metal Complexes and Nrf2 in HCC

Some metal complexes have displayed anti-cancer activities [164], among which ruthenium (Ru)-based complexes inhibited the proliferation, migration and invasion of HCC [145]. Of the two Ru complexes tested; 2,3,7,8,12,13,17,18-Octaethyl-21*H*,23*H*-porphine u(II) carbonyl (Ru1) and 5,10,15,20-Tetraphenyl-21*H*,23*H*-porphine Ru(II) carbonyl (Ru2), Ru2 exhibited potent anti-cancer activity in both in vitro and in vivo studies. The cytotoxicity of Ru2 against HepG2 cells was much higher than in L02 human normal liver cells. Besides cell growth, Ru2 inhibited migration and invasion of HepG2 cells. Ru2 triggered apoptosis through the generation of ROS. Ru2 not only decreased the expression of Nrf2 but also the downstream targets *Nqo1* and *HO1*. However, research on the anti-cancer activities of other metal-based complexes against HCC is still in its infancy.

## 15. miRs Regulation/Dysregulation of Nrf2 in HCC

**Figure 7:**
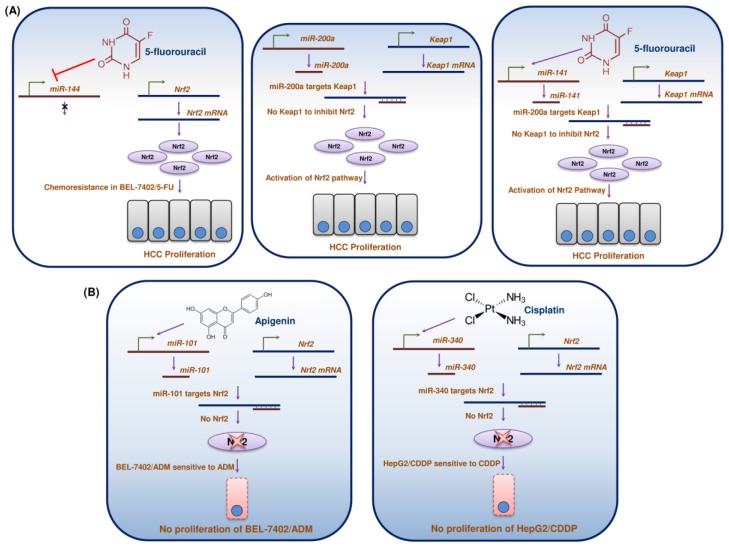
MiRs in the regulation of Nrf2 in HCC. MiRs either (**A**) activate or (**B**) suppress Nrf2, leading to chemoresistance and chemosensitization of HCC.

MiR was first identified to play a role in the regulation of development of organisms. In recent years, a multitude of roles of miRs in cancer have been explored [165]. These are short 18–25 nucleotide noncoding RNA molecules that regulate the expression of genes post-transcriptionally by binding with target mRNAs [166]. Research in the past decade unfolded the regulation of Nrf2 by miRs in HCC and may offer new approaches in the diagnostics and therapeutics for HCC (Figure 7).

Another natural compound, apigenin can chemosensitize the doxorubicin-resistant HCC cell line BEL-7402/ADM by triggering the expression of miR-101 [167]. miR-101 targets the 3′-UTR of Nrf2 and negatively regulates Nrf2, thus intensifying the sensitivity of BEL-7402/ADM to doxorubicin. miR-144 regulates chemosensitivity of HCC to 5-FU. 5-FU downregulates the expression of miR-144 and activates the Nrf2 defensive system, thereby conferring chemoresistance to BEL-7402/5-FU cells [168]. miR-144 directly interacts with the 3′-UTR of Nrf2 mRNA and inhibits the Nrf2/HO-1 pathway, which plays a vital role in the survival of BEL-7402/5-FU cells. The ectopic miR-144 expression inhibited Nrf2 and augmented the chemosensitivity of HCC cell lines to 5-FU. Yet another miR, miR-340, exhibited a similar mechanism of action in the HCC HepG2/CDDP cell line and its enhanced expression offered sensitivity against cisplatin [169]. The decreased expression of miR-340 with a concomitant increased expression of Nrf2 was detected in HepG2/CDDP cells. The luciferase reporter system revealed that miR-340 directly targets the 3′-UTR of Nrf2 mRNA in HepG2 cells. This inhibition of Nrf2 by miR-340 offered CDDP resistance to HepG2 cells. The HepG2/CDDP cells exhibited sensitivity to CDDP when these cells were transfected with miR-340 mimics, which targeted and inhibited Nrf2.

In contrast to the functions of the above discussed miRs, miR-200a promotes HCC cell growth through targeting Keap1 in the Nrf2 pathway. miR-200a negatively regulates Keap1 in both human (HepG2) and rat (FaO and RH) HCC cells when transfected with miR-200a mimics [170]. When miR-200a negatively regulates Keap1, Nrf2 may elevate the expression of its own target genes, thereby contributing to increased cell proliferation.

miR-141 expression bestows HCC resistance against 5-FU. 5-FU induces apoptosis in cancer cells through the depletion of antioxidants and hence is used as a chemotherapeutic agent against various cancers [171]. The resistance is brought by an increased expression of miR-141. The miR-141 expression was found to be elevated in HepG2/5-FU, SMMC-7721/5-FU and HuH7/5-Fu cells compared to their parental counterparts [172]. miR-141 inhibited 5-FU-induced apoptosis in HCC. miR-141 directly targets the 3′-UTR of Keap1 mRNA and thereby decreases the levels of Keap1 mRNA and protein. The suppression of Keap1 expression by miR-141 activates the Nrf2 pathway, eventually conferring resistance of HCC against 5-FU.

Nrf2 can also regulate glucose metabolism, which is essential for tumour growth [173]. The regulation of metabolic genes involves miR-1 and miR-206, which revealed the link between Nrf2 and these miRs [174]. In both tumour and non-tumour cells, the activation of Nrf2 suppressed the expression of miR-1 and miR-206 through a redox dependent mechanism. In addition, epigenetic mechanisms such as DNA methylation and acetylation in the promoter regions of miR-1 and miR-206 can regulate their expression. miR-1 and miR-206 expressions are repressed by hypoacetylation and cytosine methylation in their promoters. Histone deacetylase 4 (HDAC4) controls the acetylation of miR-1 and miR-206 promoters. miR-1 and miR-206 targets this HDAC, which in turn controls the expression of these miRs and thus establishes a regulatory feedback loop. The cysteine residues in the HDAC4 can be oxidized due to the increased ROS, eventually translocating HDAC4 into the nucleus. In tumours, persistent Nrf2 activation controls this event and thus inhibits HDAC4 activation, which thereby attenuates the expression of both miR-1 and miR-206. This attenuation of miR-1 and miR-206 targets the pentose phosphate pathway genes *glucose-6-phosphate dehydrogenase*, *6-phosphogluconate dehydrogenase*, *glycerol-3-phosphate dehydrogenase* and *transketolase*, consequently regulating glucose metabolism. Moreover, Nrf2 activity can also regulate miR-1 and miR-206 expression and thus controls glucose metabolism, ultimately leading to the progression of tumour growth. In both human liver cancers and hepatoma cell lines (HepG2 and Hep3B), miR-1 expression was reduced and its elevated expression attenuated the proliferation of hepatoma cells [175].

The above studies on miRs revealed that increased or decreased expression of miRs might serve as a therapeutic strategy to overcome liver cancer, particularly drug resistant HCC. Furthermore, miRs may serve as potential molecular markers for the diagnosis of Nrf2-stabilized cancers.

## 16. Conclusions and Future Perspectives

Nrf2 is the primary player in HCC, as evidenced from the many studies demonstrating an increased expression of Nrf2 associated with HCC. This dysregulated Nrf2 signalling promotes cellular proliferation, triggers vascularization, invasiveness and confers resistance against drugs. Hence, this master regulator could serve as a promising therapeutic target in HCC. Naturally occurring phytochemicals can modulate Nrf2 expression to cause anti-cancer effects in HCC. In addition, phytochemicals were found to reverse the chemotherapeutic resistance of HCCs and sensitized them to anti-cancer drugs. Some dietary phytochemicals are also able to modulate Nrf2 expression via miRs and targeting these miRs will open avenues for therapeutics against HCC. Cancer pharmacologists around the world are actively involved in the identification of safe and potent Nrf2 inhibitors as potential drugs against HCC. A comprehensive strategy must be framed in the development of drugs that target Nrf2 in the prevention and treatment of HCC. Molecular profiling will help in the identification of novel targets against Nrf2 in HCC. Research on epigenetic regulatory mechanisms of Keap1 and Nrf2 in HCC is still in its infancy and investigations are warranted in this area. Nrf2 can control the metabolic reprogramming that favours cellular proliferation in HCC and an approach that targets this metabolic reprogramming will shed light on the unknown targets for developing treatment options. Future investigations on the mechanisms that contribute to the Keap1-independent activation of Nrf2 will also unravel new therapeutic targets against HCC. Numerous hurdles and challenges still await scientists to unravel the complexities in the cross talk between Nrf2 and other network signalling pathways, which may pave the way to identify safe and potent Nrf2 modulators.

## Figures and Tables

**Table 1:** Phytochemicals and molecules that target different molecules and modulate the Nrf2 pathway in HCC.

Compounds	Dose and Duration	Cell Lines/Animal Model	Molecular Targets	Molecular Mechanism	Reference
Camptothecin	0.1 and 0.5 µM for 24 h	HepG2	↓GCLC, ↓GCLM, ↓NQO1, ↓HMOX-1, ↓AKR1C1, ↓AKR1C2, ↓AKR1C3	Down regulation of NRF2 suppression of ARE- dependent genes	[143]
Capsaicin	200 µM for 24 h	HepG2	↑HO-1, ↓NQO1, ↑p-AKT, ↑p-ERK, ↑NRF2	Down regulation of NQO1 triggers the production of ROS, leading to phosphorylation of AKT, ERK and ARE binding of NRF2	[127]
Glycycoumarin	10 mg and 20 mg/kg for once a day for 3 weeks	C57BL/6 mice	↑NRF2, ↑HO-1, ↑GCLC	Glycycoumarin activates NRF2 and induces autophagy via up regulation of p62 and p38	[144]
Glycycoumarin	50 µM for 24 h	HepG2	↑Nrf2, ↑HO-1, ↑GCLC, ↑p38, ↑p-ERK1/2, ↑p62, ↓KEAP1, ↑LC3-II	Glycycoumarin activates NRF2	[144]
Crotonaldehyde	50 µM for 24 h	HepG2	↑HO-1, ↑p38, ↑p-PKC-δ, ↑NRF2	Anti-apoptotic effect of crotonaldehyde induced by HO-1 through the PKC-δ-p38 MAPK-NRF2 signalling pathway	[129]
Pelargonidin Chloride	50 and 100 µM for 2 h	HepG2	↑NRF2, ↑HO-1, ↑GST, ↑NQO1, ↑CAT, ↑SOD1, ↑GPX1	Up regulation of detoxification enzymes genes through the KEAP1/NRF2 signalling pathway	[136]
Pomegranate emulsion	1 g and 10 g/kg for three times a week	Male Sprague-Dawley rats	↑GSTA2, ↑GSTA5, ↑GSTM1, ↑GSTM7, ↑GSTT1, ↑NQO1, ↑UGT1A1, ↑UGT2B17, ↑NRF2	Induction of antioxidant and phase 2 xenobiotic enzymes leading to up regulation of NRF2	[140]
Ruthenium complex	2 and 4 µM for 24 h	HepG2	↓NRF2, ↓NQO1, ↓HO-1	Suppression of NQO1 and HO-1 expression through down regulation of the Nrf2 signalling pathway	[145]

**Table 2:** Phytochemicals and molecules that sensitize resistant HCC against chemoresistance drugs.

Compounds	Drug Sensitized	Dose and Duration	Cell Lines	Mode of Nrf2 Inhibition	Molecular Targets	Reference
Apigenin	Doxorubicin	Apigenin-20 μM for 24 h Doxorubicin-2 μM for 24 h	BEL-7402/ADM	NRF2 expression was inhibited by down regulation of the PI3K/AKT pathway	↓NRF2, ↓HO-1, ↓AKR1B10, ↓MRP5	[147]
Chrysin	Doxorubicin	Chrysin-20 μM for 24 h	BEL-7402/ADM	Chrysin suppressed the activation of NRF2 and its downstream genes through inhibition of the PI3K/AKT and ERK signalling pathway	↓NRF2, ↓HO-1, ↓AKR1B10, ↓MRP5, ↓p-Akt, ↓p-ERK1/2	[148]
DMC	5-FU	DMD-5,10 and 20 μM for 24 h	BEL-7402/5-FU	NRF2 suppression, prevented NRF2 translocation and inhibited the ARE binding	↓NRF2, ↓GCLC, ↓GCLM, ↓GST, ↓GSH	[151]
Sorafenib	5-FU	Sorafenib-2 µM for 24 h 5-flurouracil-1000 μg/mL for 24 h	Bel-7402/5-FU	Sorafenib inhibited the expression of NRF2 induced by 5-flurouracil	↓NRF2, ↓MRP1, ↓MRP2, ↓MRP3	[153]
Ursolic acid	Cisplatin	Ursolic acid-2.25 μg/mL for 48 h	HepG2/DDP	Ursolic acid highly induced ROS and reduced mitochondrial membrane potential, leading to suppression of NRF2 expression and its downstream genes	↓NRF2, ↓HO-1, ↓NQO1, ↓GST	[155]
Valproic acid	Proton therapy	Valproic acid-1 mM for 2 h and 24 h	Hep3B	NRF2 expression was suppressed by NADPH oxidase activation through increased intracellular ROS level	↑PARP cleavage, ↑caspase-3 cleavage, ↓NRF2, ↓HO-1	[158]
